# Spatiotemporal Brain Dynamics of Empathy for Pain and Happiness in Friendship

**DOI:** 10.3389/fnbeh.2016.00045

**Published:** 2016-03-30

**Authors:** Yiwen Wang, Juan Song, Fengbo Guo, Zhen Zhang, Sheng Yuan, Stephanie Cacioppo

**Affiliations:** ^1^School of Humanities and Social Sciences, Institute of Psychological and Cognitive Sciences, Fuzhou UniversityFuzhou, China; ^2^Academy of Psychology and Behavior, Tianjin Normal UniversityTianjin, China; ^3^Department of Psychiatry and Behavioral Neuroscience, Biological Sciences Division, The University of Chicago Pritzker School of MedicineChicago, IL, USA; ^4^High-Performance Electrical Neuroimaging (HPEN) Laboratory, The University of ChicagoChicago, IL, USA

**Keywords:** electrical neuroimaging, happiness, sense of self, friendship, interpersonal processes, mirror mechanism, dyads, social neuroscience

## Abstract

**Background:** Although a large number of functional magnetic resonance imaging (fMRI) studies have investigated the neural bases of empathy, little is known about its spatiotemporal dynamics or its modulation by the level of friendship between the observer and the agent who is being hurt. Moreover, most of the previous studies on empathy have focused on empathy for pain rather than empathy for positive emotions, such as happiness. In the present study, we addressed this question by investigating the spatiotemporal brain dynamics of two different kinds of empathy (empathy for pain, empathy for happiness) with a behavioral priming empathy task involving two different level of primes (a close friend, a stranger).

**Method/Principal Findings:** Electrical brain activity and behavioral data were analyzed from 30 subjects (12 males and 18 females). Half of the subjects performed a behavioral task on empathy for pain task (EPT), while the other half performed a behavioral task on empathy for happiness task (EHT). In each task, participants viewed prime photographs of either: (1) a stranger; or (2) a close friend (*primes*) followed by target photographs showing either a hand being hurt (or not; *targets* in the EPT), or a hand in happy circumstances (or not; *targets* in the EHT). In each task, participants were asked to judge the *target* situation and report whether they could feel the pain (in EPT) or the happiness (in the EHT), as a function of the primes i.e., either from the close friend’s or from the stranger’s perspective. Although our behavioral results didn’t reveal any explicit differences among the different types of primes within each task, our electrophysiological results showed variations as a function of the primes. First, a early smaller N110 amplitude for pain was observed in the anterior prefrontal cortex during the friend prime condition compared to the stranger prime condition. No similar early effects were found for happiness. On the other hand, both empathy for happiness (EHT) and empathy for pain (EPT) elicited later differences. In the EPT, the friend prime elicited a larger late positive potential (LPP) than the stranger prime. In the EHT, the friend prime elicited a larger N250, a smaller P300, and a smaller LPP than the stranger prime.

**Conclusions:** Taking the perspective of a close friend (as a prime stimulus) does have a dual-stage effect on empathy that is characterized by an early modulation for pain and later modulations for both pain and happiness. The early differences between friend and stranger primes for pain (but not for happiness) suggest that empathy for pain is an automatic process that has been socially learned and passed among friends. On the other hand, the later differences observed between stranger and friend prime suggest that additional cognitive appraisal take place for both pain and happiness. Our results suggest that it takes more cognitive attentional efforts to judge a stranger’s happiness than a friend’s happiness, whereas the opposite is true for pain. These findings open new avenues toward a better understanding of the empathic mind.

## Introduction

Empathy, the capacity to understand and share feelings with others, plays a crucial role in human social communication, connection, and interaction (Decety and Jackson, 2004). This skill has been proposed to be particularly important in the mediation of the development and acquisition of appropriate social behaviors on a daily basis (de Vignemont and Singer, 2006; Li and Han, 2010). Over the past decade, a growing body of neuroimaging studies using functional magnetic resonance imaging (fMRI) have explored the neural underpinnings sustaining the component processes of empathy. These studies used various stimuli (such as faces, bodies) and/or various priming paradigms depicting people in pain (e.g., Jackson et al., 2005, 2006; Ogino et al., 2007; Cheng et al., 2007; Gu and Han, 2007a; Lamm et al., 2007a,b; Morrison and Downing, 2007; Morrison et al., 2007; Saarela et al., 2007; Yamada and Decety, 2009; Fan et al., 2011).

Overall, these fMRI results revealed that imagining or seeing others’ body or facial expressions in pain involves the re-activation of three main neural pathways: (i) the pain network (e.g., Cacioppo et al., 2005, 2013a; Jackson et al., 2006; Akitsuki and Decety, 2009); (ii) the emotional network (Botvinick et al., 2005; Jackson et al., 2005, 2006; Singer et al., 2006; Gu and Han, 2007a; Fan et al., 2011); and (iii) the sensorimotor integration network (Avenanti et al., 2005, 2006; Bufalari et al., 2007; Gu and Han, 2007b; Valeriani et al., 2008; Fan et al., 2011). The common recruitment of a cortico-limbic network (possibly including the human mirror neurons) in these three networks reinforces the hypothesis that empathy includes two main components: (1) an affective response to another person when sharing another person’s emotional states; and (2) a cognitive response when taking the perspective of that other person. The affective component of empathy activates the dorsal anterior cingulate cortex, limbic system, and anterior insula, while the cognitive component of empathy activates the ventro-medial, medial, and dorso-medial prefrontal cortices, the posterior superior temporal sulcus (pSTS), the temporal poles, the posterior cingulate cortex, and the precuneus (Decety and Jackson, 2004; Decety and Cacioppo, 2012).

In the last few decades, significant advances have been made in our understanding of the neural correlates of empathy, but these have been mainly based on studies in which people have been looking at stimuli strictly related to pain. Rare are the studies investigating the neural bases of empathy for positive emotions (Jabbi et al., 2007; Takahashi et al., 2009; Morelli and Lieberman, 2013). While Morelli and Lieberman have attempted to investigate the neural differences between automatic and non-automatic attentional processes during three types of empathy (empathy for: (1) happiness; (2) sadness; and (3) anxiety), they did not investigate the specific neural correlates (nor the spatiotemporal dynamics) of each type of empathy. The lack of neuroimaging research on the empathy for positive emotions (e.g., happiness) is a real gap in our literature. Given that we spend much of our lifetime interacting with others in a positive (rather than negative) way, there is a real need to better understand the functional and spatiotemporal brain dynamics during empathy for positive interactions. This constitutes a strong rational for the present study.

Despite the fact that significant advances in our understanding of the neural bases of empathy have been made in the last few decades, these have been largely based on studies in which people have been considered as strictly isolated entities. For example, studies on empathy for pain typically examine how participants judge (or share) a visual scene in which a stranger (rather than a close friend) is being hurt. Little is known, however, about the neural bases of empathy for pain (or happiness) when a close friend is being hurt (or rewarded). The present paper addresses this question. We hypothesized that the brain network sustaining empathy (including the mirror neuron system) should be activated faster when a close friend (rather than a stranger) is involved in the visual scene. Our hypothesis was based on a growing body of studies suggesting that the social closeness/distance between one’s self and others (such as the similarity between one’s self and others/target, Batson et al., 1997a,b; the likability of others/targets, Kozak et al., 2006; the group of the observer and the target, Stürmer et al., 2006; Yabar et al., 2006; and the pair bond between the observer and the target/others, Cheng et al., 2010) may modulate empathy (Avenanti et al., 2005, 2006; Singer et al., 2006; Bufalari et al., 2007; Engert et al., 2014; Gleichgerrcht and Decety, 2014). Furthermore, we assumed that the areas involved in differentiating one’s self from others should be activated more strongly for a stranger than for a close friend. Our hypothesis is based on Cheng et al.’s (2010) study that combined fMRI with a priming paradigm presenting photographs of a participants’ loved one and photographs of strangers before a behavioral task evaluating the participants’ empathy for pain. Cheng and colleagues showed that taking the perspective of a stranger involved a signal increase in the right temporo-parietal junction (TPJ) and the superior frontal gyrus (two areas known to be involved in self-other discrimination), whereas taking the perspective of a loved one increased brain activity in the anterior cingulate cortex and insula (two areas involved in self-other integration; e.g., Craig, 2009; Cacioppo et al., 2012, 2013a,b). As expected, the closer the participants felt with their loved one, the greater was the deactivation in the right TPJ. When participants were imagining the perspective of a stranger, a negative correlation was found between the right TPJ and the insula, while a positive correlation was found with the superior frontal gyrus (Cheng et al., 2010).

Finally, rare are the studies that investigate the spatiotemporal dynamics of empathy in the human brain (Li and Han, 2010; Decety and Cacioppo, 2012). The low temporal resolution of fMRI techniques limits such investigation. Studies using high-temporal resolution (at the millisecond range), such as high-density electrical neuroimaging, can, on the other hand, identify patterns of fast communication between regions that slower contrast analyses may not detect (Cacioppo et al., 2014; Cacioppo and Cacioppo, 2015; Cacioppo, 2016). To date, only a few event-related potentials (ERPs) studies have investigated empathy for pain (Fan and Han, 2008; Han et al., 2008; Decety and Cacioppo, 2012; Meng et al., 2012; Yoder and Decety, 2014). For instance, Fan and Han (2008) recorded electrical brain activity from healthy subjects while they performed a judgment task on pain. Fan and Han’s (2008) main results showed early and late neural responses of empathy for pain. More precisely, an early ERP differentiation was found between painful and neutral stimuli over the frontal electrodes at 140 ms post-stimulus onset (Fan and Han, 2008). A later ERP component was also observed over the central-parietal electrodes around 380 ms post-stimulus onset. This difference was more salient over the left than the right hemisphere (Fan and Han, 2008).

Using high-density electrical neuroimaging, Decety and Cacioppo (2012) also found an early component at 62 ms post-stimulus onset over the right posterior temporal sulcus area and two later components around 122 ms (with a brain source estimated in the amygdala area) and 182 ms (in the ventromedial prefrontal cortex) post-action onset. These findings provided the first evidence that emotional processing may occur before any cognitive inferences occur on the morality/empathy of a scene (Decety and Cacioppo, 2012). Although these findings shed light on the spatiotemporal brain dynamics of the component processes underlying empathy for pain, little is known about the factors modulating each ERP component during other types of empathy (e.g., empathy for happiness). This is another rational for the present study.

In the present study, we investigated the brain mechanisms and time course of different types of empathy by combining electrophysiological recordings with a behavioral priming empathy task that involved two types of emotions (negative and positive emotions) and two types of primes (a close friend and a stranger).

## Materials and Methods

### Participants

A total of 34 undergraduate students initially volunteered to participate in this study. Because of poor signal-to-noise ratio in the electrophysiological data, data from four subjects were removed from the analyses (two who performed the empathy for pain task and two who performed the empathy for happiness task). The 30 remaining subjects (mean age = 20 years; range = 19–23 years; 12 males and 18 females) were included in the final analyses. All participants were right-handed, had normal or corrected-to-normal vision, and reported no history prior brain damage. Informed written consent was obtained from all participants prior to electroencephalogram (EEG) recordings. The experiment was in accordance with the ethical principles of Declaration of Helsinki.

### Procedure

Subjects were randomly assigned to perform either the empathy for pain task (EPT) or the empathy for happiness task (EHT). Half of the participants (6 males and 9 females) performed the EPT, which involved either target hands being hurt (pain condition) or not being hurt (control condition I). The other half (6 males and 9 females) performed the EHT, which included either target hands being in socially happy circumstances (e.g., picking up money; happy condition) or not (control condition II).

In each behavioral task, the subjects performed two blocks in which emotional trials and neutral trials were mixed. Two types of prime (stranger and close friend) were counterbalanced in each block.

In the EPT, each target stimulus was presented twice: once after the stranger prime and once after the friend prime. Each subject viewed a total of 352 trials.

In the EHT, a similar procedure was used. Each target stimulus was presented three times: once after the stranger prime and once after a friend prime. Each subject viewed a total of 240 trials.

### Stimuli

**Figure 1:**
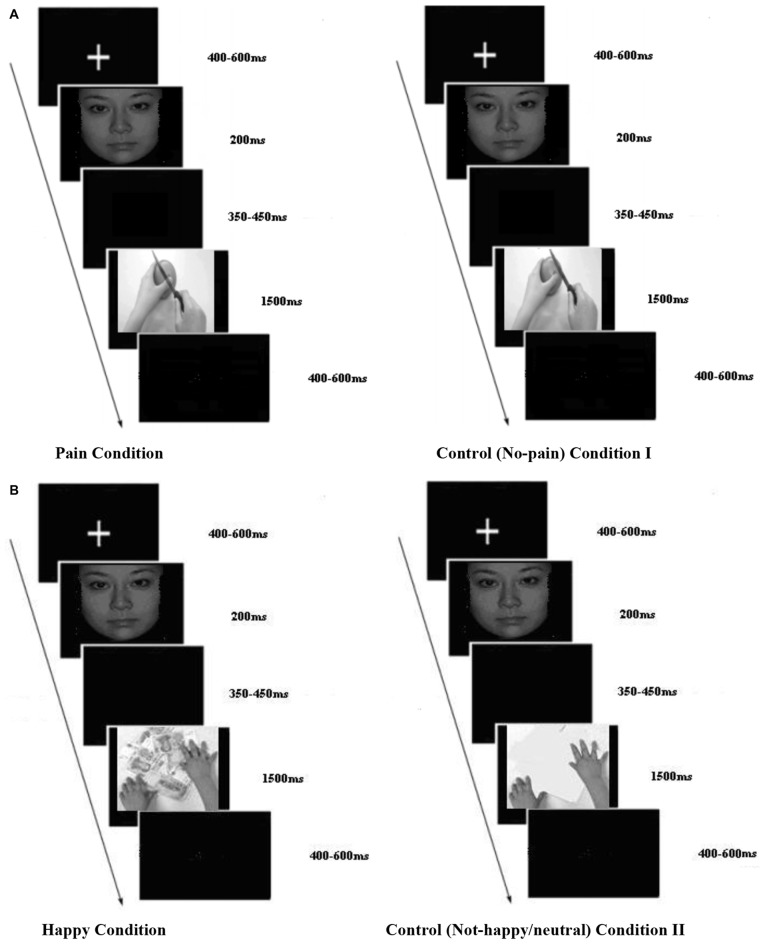
**Experimental paradigm. (A,B)** Experimental paradigm for empathy for pain task (EPT) and empathy for happiness task (EHT). A “+” was presented for 400–600 ms randomly, then after 100 ms blank the facial pictures were showed for 200 ms. Then there was a random blank changing between 350 and 450 ms. Then, the target pictures were presented for 1500 ms, and followed by an inter-trial interval that varied randomly between 400–600 ms. **(A)** EPT sample trial and **(B)** EHT sample trial.

A total of 130 photographs were used in this study. Two of these photographs (a face of a stranger and a face of a close friend of the participant) were used as primes. All the other photographs were used as target stimuli. In the EPT, 44 of the target stimuli were photographs of hands in a painful situation (pain condition) and 44 other photographs showed hands in a non-painful situation (control condition I; see Figure 1A). In the EHT, 20 of the target stimuli were photographs of hands interacting with different objects in a happy situation (e.g., grasping money) and 20 other photographs showed hands interacting with a neutral situation (control condition II; see Figure 1B). The combination of faces and hands with realistic target situations constitutes an improvement compared to previous studies as they provide a more ecological context. To control for visual features across the stimuli, we presented all photographs in black and white and all photographs had the same size (370 × 290 pixels).

### Stimulus Selection

For the EPT, 22 undergraduate students (mean age = 20.3 years; *SD* = 0.9 years) evaluated the 88 photographs by judging the intensity of pain of each one of the photographs on a 4-point scale from 0 (non painful) to 4 (very painful). Results revealed a mean rating of 2.2 (*SD* = 0.8). For the EHP, 21 undergraduate students (mean age = 20.3 years; *SD* = 0.9 years) evaluated the 40 photographs by judging the intensity of happiness of each one of the photographs on a similar 4-point scale from 0 (non-happy/neutral) to 4 (very happy). Results revealed a mean rating of 2.3 (*SD* = 0.8).

A total of 30 prime stimuli of portrait photographs of strangers were chosen from Chinese Affective Picture System (CAPS; Bai et al., 2005) and were matched for age, valence, facial attractiveness, and the arousal level with the portrait photographs of the subjects’ friend. One hundred undergraduate students (50 men, 50 women; mean age = 22.6) evaluated the neutral intensity of the CAPS on a scale from 1 (the weakest) to 9 (the strongest). On average, the intensity of the neutral face photographs was evaluated at 5.75, and the mean accuracy of the neutral face pictures was 84.45%.

For the prime stimuli of a close friend, participants were asked to bring a gender-matched close friend with them to the laboratory where the experimenter took a portrait photograph of both the participant and their close friend, prior conducting the combined electrophysiological and behavioral experiment.

### Experimental Paradigm and Participants’ Instruction

Each trial began with a central presentation of a random fixation cross between 400 and 600 ms. The prime stimulus was then presented for 200 ms. Then, a blank screen was presented for 350–450 ms (determined randomly). Finally, the target stimulus was presented for 1500 ms. The interval between trials was randomly presented between 400 and 600 ms (Figures 1A,B). Each block began with a 3 s screen that included the participants’ instruction.

Participants were asked to pay attention to the center of the screen signaled by “+” and to the subsequent photographs. They were told that they would first see either a photograph of their friend or a stranger, followed by a picture of hands of the person displayed in the previous photograph. For each trial, subjects were asked to indicate as quickly and as accurately as possible whether (or not) each photograph of hands represented a painful situation (EPT) or happy situation (EHT). Participants received monetary compensation for their participation.

### Questionnaires

As in previous studies on empathy, participants were asked to complete the Interpersonal Reactivity Index (IRI)-C questionnaire, which is a revised version of the IRI (Davis, 1980) with specific sensitivity to the nuances of the Chinese culture (Zhang et al., 2010; Zhan unpublished). IRI-C contains 22 5-point Likert scale items grouped in four subscales: (1) Perspective Taking (PT); (2) Empathic Concern (EC); (3) Personal Distress (PD); and (4) Fantasy (F). The IRI-C had poor internal consistency for EC subscale (0.72 for PT; 0.62 for F; 0.53 for EC; 0.76 for PD) and test-retest reliability (0.70 for PT; 0.74 for F; 0.63 for EC; 0.66 for PD; Zhang et al., 2010; Zhan, unpublished). Participants were also asked to report the duration of their friendship with the close friend they brought to the laboratory.

### Electrophysiological Data Acquisition and Pre-Processing

The EEG was continuously recorded from 66 scalp electrodes that were mounted on an elastic cap in accordance to the extended 10–20 system, with the addition of two mastoid electrodes. The electrode at the left mastoid was used as recording reference and transformed to the average of two mastoids offline. Our result is dependent on the reference of the average value right and left mastoids. The electrode impedance was kept less than 5 kΩ. Eye blinks and vertical eye movements were monitored with electrodes located above and below the left eye. The horizontal electro-oculogram was recorded from electrodes placed 1.5 cm lateral to the left and right external canthi. The EEG was amplified (band pass: 0.05–100 Hz) and digitized at a sampling rate of 1000 Hz.

The EEG data were re-referenced to linked mastoids offline. A band-pass filter between 0.05 and 30 Hz was then applied. EEG data were segmented offline into 1200 ms epochs spanning from 200 ms pre-stimulus (target) to 1000 ms post-stimulus onset. The data were baseline corrected from −200 ms to 0 ms. Trials contaminated by eye blinks, eye movements, or muscle potentials exceeding ± 75 μv at any electrode were excluded from the average.

### Brain Source Estimations

To investigate the potential brain sources of our ERP components we used sLORETA (Pascual-Marqui, 2002,^1^).This software reports Montreal Neurological Institute (MNI) coordinates. The head model for the inverse solution uses the realistic electrode coordinates (Jurcak et al., 2007) and the head model (an electric potential lead field matrix (Fuchs et al., 2002) computed with the boundary element method applied to the MNI152 template (Mazziotta et al., 2001). Brain estimations were conducted on the whole scalp recording electrodes after statistical analyses were performed on the ERP components. We restricted the sLORETA (Pascual-Marqui, 2002) brain source estimations to specific time windows that showed significant ERP differences in our ERP analyses. The analyses were performed with normalized data, and applied baseline correction.

### Statistical Analyses

#### Behavioral Analyses

A 2 × 2 analysis of variance (ANOVA) with prime type (friend; stranger) and target type (emotional; neutral) as within-subjects was conducted for reaction times (RT) and accuracy rate (AR) in EPT and EHT, respectively. Correlation analyses were also conducted between IRI-C and behavioral data (see Supplementary Material). To investigate whether the two groups of subjects had similar behavioral performance (RT and/or AR) in response to each type of prime, we also performed a 2 × 2 × 2 repeated-measures ANOVA with type of task (EPT; EHT) as between-subjects factor, and target type (emotional; neutral) and prime type (friend; stranger) as within-subjects factor. To make it comparable, the number of trials in EPT for behavioral analysis was matched with the number of trials in EHT. The average data of first 60 trials under each condition (friend primed pain, friend primed no pain, stranger primed pain, stranger primed no pain) in EPT was matched with that of 60 trials under each condition in EHT (totally four conditions of 240 trials).

#### Electrophysiological Analyses

We performed a three-way repeated-measures ANOVA with prime type (stranger face; friend face), target type (emotional; neutral), and electrode site (F3, FZ, F4, FC3, FCZ, FC4, C3, CZ, C4, CP3, CPZ, CP4, P3, PZ, P4) as within-subject factors for the EPT and EHT, respectively. We also performed a four-way repeated-measures ANOVA with prime type (stranger; friend), target type (emotional; neutral), anterior-posterior position (frontal; fronto-central; central; central-parietal; parietal) and laterality (left; midline; right) as within-subject factors for the EPT and EHT, respectively. Grand-averaged ERPs were computed separately for each task (EPT and EHT). As in previous studies on empathy for pain (e.g., Fan and Han, 2008; Decety et al., 2010), we analyzed the ERP components of three components known to be important in empathy tasks i.e., (1) N110 (peak amplitude between 80–150 ms) in fronto-central area; (2) P300 (peak amplitude between 300–400 ms) in posterior area; and (3) late positive potential (LPP; mean amplitude at 400–800 ms) in posterior area. In addition, because N2 (peak amplitude between 200–300 ms) has been shown to be relevant to emotion regulation and social emotion reappraisal (Canli et al., 2009; Lamm and Lewis, 2010), we also analyzed N250 component. The Greenhouse-Geisser method was applied to the corrected *p*-values to account for violations to the ANOVA assumption of sphericity.

## Results

### Questionnaires

All subjects reported being in a friendship for at least 6 months. In EP task, the mean duration of friendship was 27 months, while the mean duration in EH task was 26 months. Independent *t*-test showed no significant difference between the two groups (*Mean*_EPT_ = 27 months and *Mean*_EHT_ = 26 months; *t*_(28)_ = 0.306, *p* = 0.762).

Results from the IRI-C revealed the total scores (±SD) of the four dimensions were the following: 13.07 ± 3.75 (PT), 17.27 ± 4.40 F, 16.00 ± 3.21 (EC), and 10.53 ± 5.42 (PD). Mean scores and standard deviations (SD) of IRI-C at each dimension were 2.64 ± 0.77 (PT), 2.81 ± 0.71 F, 2.70 ± 0.54 (EC) and 2.09 ± 1.12 (PD). Mean scores were used for correlation statistical analysis.

### Behavioral Performance

#### Empathy for Pain Task (EPT)

Table 1 displays mean RTs and response ARs for each EPT experimental condition.

**Table 1:** **Behavioral results for each experimental condition**.

Condition	RT (ms)	AR (%)
**Empathy for pain**
Friend prime pain target	771 ± 71	81.67 ± 3.99
Friend prime non-pain target	805 ± 74	80.47 ± 3.25
Stranger prime pain target	765 ± 77	80.73 ± 4.25
Stranger prime non-pain target	799 ± 81	81.27 ± 3.67
**Empathy for happiness**
Friend prime happy target	718 ± 85	95.32 ± 6.62
Friend prime non-happy target	705 ± 91	92.98 ± 7.79
Stranger prime happy target	705 ± 80	96.49 ± 6.26
Stranger prime non-happy target	709 ± 82	92.28 ± 8.84

*RT, reaction times in ms; AR, accuracy rate in %*.

Two-way repeated-measures ANOVA of response time with prime type (friend; stranger) and target type (pain; control I) indicated that there was a significant effect of target type (*F*_(1,14)_ = 8.959, *p* = 0.010, *η*^2^ = 0.390), with RTs for pain targets being faster than those for no pain targets. There was no significant effect of prime type (*F*_(1,14)_ = 1.850, *p* = 0.195, *η*^2^ = 0.117) or interaction effect (*F*_(1,14)_ < 0.001, *p* = 0.995, *η*^2^ < 0.001) for RTs. There were no significant results for ARs: There was no significant main effect of prime type (*F*_(1,14)_ = 0.011, *p* = 0.918, *η*^2^ = 0.010), or main effect of target type (*F*_(1,14)_ = 0.054, *p* = 0.819, *η*^2^ = 0.004), or interaction effect between prime and target type (*F*_(1,14)_ = 1.907, *p* = 0.189, *η*^2^ = 0.120).

#### Empathy for Happiness Task (EHT)

Table 1 displays mean RTs and response ARs for each EHT experimental condition. Two-way repeated-measures ANOVA of RTs with prime type (friend, stranger) and target type (happy, control II) showed no significant results. There was no significant main effect of prime type (*F*_(1,14)_ = 0.903, *p* = 0.358, *η*^2^ = 0.061) and no main effect of target type (*F*_(1,14)_ = 0.148, *p* = 0.706, *η*^2^ = 0.010). Similarly, a two-way repeated-measures ANOVA of ARs with prime type (friend, stranger) and target type (happy, control II) showed no significant results. There was no significant main effect of prime type (*F*_(1,14)_ = 0.126, *p* = 0.728, *η*^2^ = 0.009), or main effect of target type (*F*_(1,14)_ = 1.485, *p* = 0.243, *η*^2^ = 0.096).

### EPT vs. EHT

#### Reaction Times

Our results revealed a significant interaction between target type and task type (*F*_(1,28)_ = 7.862, *p* = 0.009, *η*^2^ = 0.219). Further analyses revealed that, in emotional trials, mean RTs (±SE) for empathy for pain were significantly longer (792 ± 22 ms) than those for empathy for happiness trials (711 ± 22 ms; *F*_(1,28)_ = 7.012, *p* = 0.013, *η*^2^ = 0.200). In neutral trials, mean RTs (±SE) for empathy for no pain trials were significantly longer (835 ± 21 ms) than those for empathy for no happy trials (707 ± 21 ms; *F*_(1,28)_ = 17.977, *p* < 0.001, *η*^2^ = 0.391).

In addition, our between-subject factor analysis revealed significant RT differences (*F*_(1,28)_ = 12.839, *p* = 0.001, *η*^2^ = 0.314), with mean RTs (±SE) for empathy for pain (814 ± 21 ms) being longer than those for empathy for happiness (709 ± 21 ms). Within-subject factor analysis also showed significant RT differences of target type (*F*_(1,28)_ = 5.415, *p* = 0.027, *η*^2^ = 0.162), with mean RTs (±SE) for emotional targets being shorter (752 ± 15 ms) than those for neutral targets (771 ± 15 ms).

##### Accuracy

For ARs, our results revealed an interaction between target type, prime type, and task type (*F*_(1,28)_ = 5.618, *p* = 0.025, *η*^2^ = 0.167). Further analyses revealed a significant effect between task type under stranger primed emotional trials (*F*_(1,28)_ = 6.653, *p* = 0.015, *η*^2^ = 0.192), which indicated that ARs (±SE; 96.49 ± 1.56) of happy trials were higher than those of pain trials (90.77 ± 1.56). Further analyses revealed no significant differences between task type under friend primed emotional trials (*F*_(1,28)_ = 1.613, *p* = 0.215, *η*^2^ = 0.054).

#### Correlation Analyses of Behavioral Data

No significant correlations were observed between IRI-C and the EHT behavioral data (RT and AR). Because no behavioral correlations were found between EHT and IRI-C, no further analyses were between electrophysiological data and IRI-C.

In the EPT, there was also no significant correlations between the four dimensions of the IRI-C and the RT. Analyses between ARs and the four dimensions of the IRI-C revealed, however, a positive correlation between the fantasy dimension and the response AR for the friend prime (*r* = 0.662, *p* = 0.004) and stranger prime (*r* = 0.428, *p* = 0.056). There was also a positive correlation between EC and stranger prime (*r* = 0.493, *p* = 0.031). These positive correlations were observed in the pain condition. For the “no pain” condition, negative correlations were observed between fantasy and friend prime (*r* = −0. 446, *p* = 0.048), and between PD and friend prime (*r* = −0.475, *p* = 0.037). Finally, there was a negative correlation between PT and friend prime (*r* = −0.528, *p* = 0.021).

## Electrophysiological Data

In EPT, the number of average accepted trials were 64 under friend primed pain condition, 65 under friend primed no pain condition, 63 under stranger primed pain condition and 65 under stranger primed no pain condition. In EHT, the number of average accepted trials were 43 under friend primed happy condition, 42 under friend primed no happy condition, 44 under stranger primed happy condition and 40 under stranger primed no happy condition.

### Empathy for Pain Task (EPT; Figure 2)

#### N110 (Peak Amplitude between 80–150 ms)

**Figure 2:**
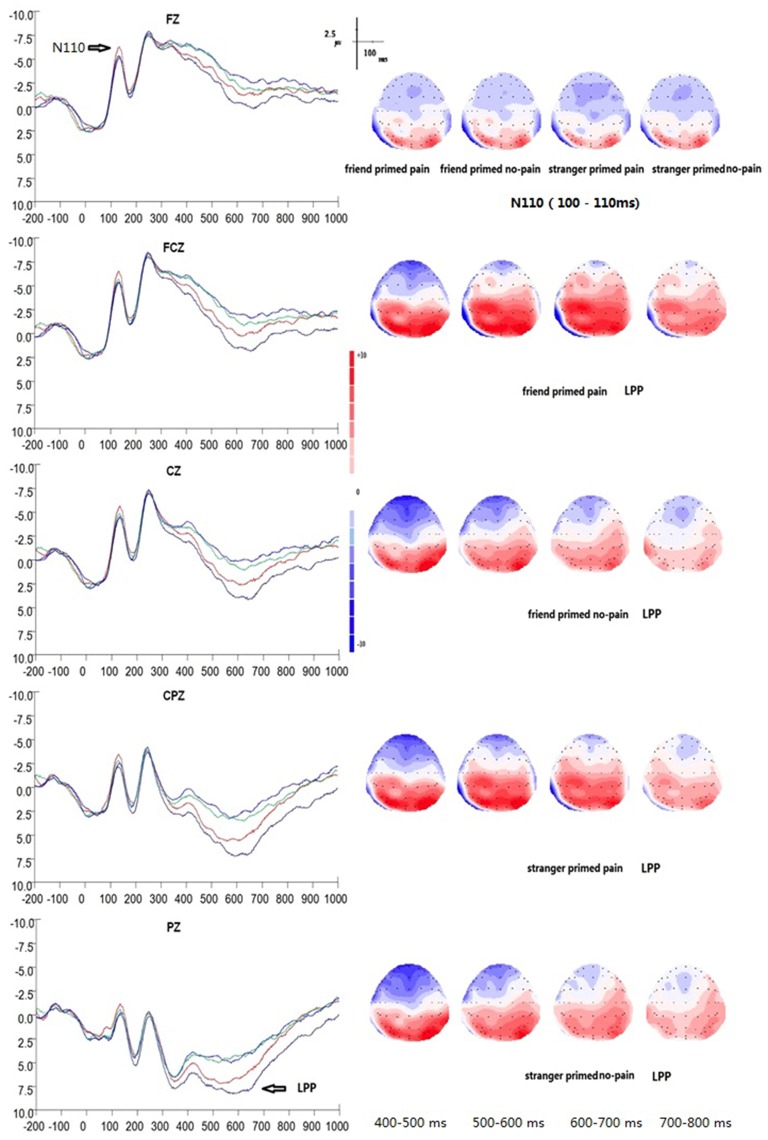
**Grand average event-related potential (ERP) waveforms and 2D mapping of empathy for pain.** Fz, FCz, Cz, CPz and Pz for friend primed pain (dark blue lines), friend primed no pain (light blue lines), stranger primed pain (red lines), and for stranger primed no pain (green lines).

In EPT, the peak amplitudes of N110 between 80–150 ms showed significant main effects of electrodes (*F*_(14,196)_ = 21.167, *p* = 0.000, *η*^2^ = 0.602). The maximum peak occurred at electrode of FCZ (*M* = −6.23 μv, *SE* = 1.35 μv, */M/* = 6.23 μv), whereas the minimum amplitude was at P3 (*M* = 0.14 μv, *SE* = 0.77 μv, */M/* = 0.14 μv). There was a significant interaction of prime type × target type (*F*_(1,14)_ = 5.622, *p* = 0.033, *η*^2^ = 0.287), suggesting that the stranger prime elicited significant larger N110 amplitude of pain target (*M* = −4.50 μv, *SE* = 1.06 μv, */M/* = 4.50 μv) than that of the friend prime (*M* = −3.52 μv, *SE* = 1.02 μv, */M/* = 3.52 μv; *F*_(1,14)_ = 6.767, *p* = 0.021, *η*^2^ = 0.326). On the other hand, for no-pain targets, there as no significant difference between stranger prime condition (*M* = −3.82 μv, *SE* = 1.06 μv, */M/* = 3.82 μv) and friend prime condition (*M* = −3.80 μv, *SE* = 1.07 μv, */M/* = 3.80 μv; *F*_(1,14)_ = 0.002, *p* = 0.963, *η*^2^ < 0.001). There was also no significant interaction between electrodes and prime type (*F*_(14,196)_ = 0.175, *p* = 0.917, *η*^2^ = 0.012), electrodes and target type (*F*_(14,196)_ = 1.821, *p* = 0.128, *η*^2^ = 0.115), or electrodes, target type, and prime type (*F*_(14,196)_ = 0.483, *p* = 0.740, *η*^2^ = 0.033).

A 2 × 2 × 3 × 5 repeated measures ANOVA was performed with prime type, target type, laterality, and anterior to posterior position. The results showed a significant main effect of anterior to posterior brain areas at the level of N110 (*F*_(4,56)_ = 26.762, *p* < 0.001, *η*^2^ = 0.657). There was also a main effect of laterality factor (*F*_(2,28)_ = 8.173, *p* = 0.002, *η*^2^ = 0.369), and a significant interaction between prime type × target type (*F*_(1,14)_ = 5.622, *p* = 0.033, *η*^2^ = 0.287), suggesting that the stranger prime (*M* = −4.50 μv, *SE* = 1.06 μv, */M/* = 4.50 μv) elicited a larger N110 amplitude for pain target than that of the friend prime (*M* = −3.52 μv, *SE* = 1.02 μv, */M/* = 3.52 μv). Finally, we found an interaction between prime type and anterior to posterior position factor (*F*_(2,28)_ = 4.015, *p* = 0.033, *η*^2^ = 0.223), and an interaction between laterality, and anterior to posterior position factors (*F*_(8,112)_ = 3.416, *p* = 0.036, *η*^2^ = 0.196).

#### N250 (Peak Amplitude between 200–300 ms)

In EPT, N250 peaked on average at 249 ms (*SD* = 4 ms). There was no significant differences, except for the main effect of electrodes (*F*_(14,196)_ = 33.333, *p* = 0.000, *η*^2^ = 0.704), which showed maximum values at FCZ (*M* = −9.91 μv, *SE* = 1.62 μv, */M/* = 9.91 μv) and minimum values at P4 (*M* = 2.98 μv, *SE* = 1.23 μv, */M/* = 2.98 μv). There was no significant effect of prime type (*F*_(1,14)_ = 0.899, *p* = 0.359, *η*^2^ = 0.060) or target type (*F*_(1,14)_ = 0.005, *p* = 0.945, *η*^2^ < 0.001). Similarly, there was no interaction between prime type and electrodes (*F*_(14,196)_ = 0.325, *p* = 0.788, *η*^2^ = 0.023), or between target type and electrodes (*F*_(14,196)_ = 2.959, *p* = 0.060, *η*^2^ = 0.174). There was no interaction between prime type and target type (*F*_(1,14)_ = 0.725, *p* = 0.409, *η*^2^ = 0.049), or between the three factors as well (*F*_(14,196)_ = 1.150, *p* = 0.343, *η*^2^ = 0.076).

A 2 × 2 × 3 × 5 repeated measures ANOVA was performed. The results show a significant main effect of anterior to posterior position at the level of N250 (*F*_(4,56)_ = 37.560, *p* < 0.001, *η*^2^ = 0.728). There was also a main effect of laterality (*F*_(2,28)_ = 26.312, *p* < 0.001, *η*^2^ = 0.653), a significant interaction between target type and laterality (*F*_(2,28)_ = 13.785, *p* < 0.001, *η*^2^ = 0.496), and a significant interaction between laterality and anterior to posterior position (*F*_(8,112)_ = 4.284, *p* = 0.008, *η*^2^ = 0.234).

#### P300 (Peak Amplitude between 300–400 ms)

In EPT, the P300 peaked on average at 369 ms (*SD* = 23 ms). ANOVAs of the peak ERP amplitudes between 300–400 ms showed a significant main effect of electrodes (*F*_(14,196)_ = 49.578, *p* < 0.001, *η*^2^ = 0.780). The maximum peak occurred at electrode P4 (*M* = 10.35 μv, *SE* = 1.55 μv), whereas the minimum amplitude was at P3 (*M* = −4.45 μv, *SE* = 1.29 μv). There was a significant interaction between electrodes × target type (*F*_(14,196)_ = 3.701, *p* = 0.011, *η*^2^ = 0.209). Furthermore, there was a significant difference between pain and no pain responses over electrodes CP1 (*F*_(1,14)_ = 6.61, *p* = 0.022), P1 (*F*_(1,14)_ = 6.77, *p* = 0.021) and PZ (*F*_(1,14)_ = 4.84, *p* = 0.045). At CP1, P300 was smaller for the stranger prime condition (*M* = 4.51 μv, *SE* = 1.35 μv) than that of the friend prime condition (*M* = 5.61 μv, *SE* = 1.35 μv). At P1, P300 was smaller for the stranger prime condition (*M* = 9.46 μv, *SE* = 1.56 μv) than that for the friend prime condition (*M* = 10.39 μv, *SE* = 1.66 μv). At PZ, P300 was smaller for the stranger prime condition (*M* = 8.09 μv, *SE* = 1.80 μv) than that for the friend prime condition (*M* = 8.98 μv, *SE* = 1.88 μv). There was no significant main effect of prime type (*F*_(1,14)_ = 0.479, *p* = 0.500, *η*^2^ = 0.033) or target type (*F*_(1,14)_ = 2.128, *p* = 0.167, *η*^2^ = 0.132). There was also no interaction between prime type and target type (*F*_(1,14)_ = 3.221, *p* = 0.089, *η*^2^ = 0.192). There was also no interaction between prime type and electrodes (*F*_(14,196)_ = 0.693, *p* = 0.553, *η*^2^ = 0.047). Finally, there was no significant interaction among the three factors (*F*_(14,196)_ = 0.251, *p* = 0.864, *η*^2^ = 0.018).

A 2 × 2 × 3 × 5 repeated measures ANOVA with prime type, target type, laterality, and anterior to posterior position showed a main effect of anterior to posterior position at the level of P300 (*F*_(4,56)_ = 59.816, *p* < 0.001, *η*^2^ = 0.810). There was also a significant main effect of laterality (*F*_(2,28)_ = 12.716, *p* < 0.001, *η*^2^ = 0.476), and an interaction between target type and laterality (*F*_(2,28)_ = 16.748, *p* < 0.001, *η*^2^ = 0.545).

#### LPP (Mean Amplitude at 400–800 ms)

ANOVAs of the mean ERP amplitudes recorded at the fronto–parietal electrodes showed a significant main effect of target type (*F*_(1,14)_ = 19.255, *p* = 0.001, *η*^2^ = 0.579), suggesting that LPP was much larger in the pain condition (*M* = 2.47 μv, *SE* = 1.21 μv) than in the control condition (*M* = 0.75 μv, *SE* = 1.29 μv). A significant interaction between electrodes and target type was also observed (*F*_(14,196)_ = 10.734, *p* = 0.000, *η*^2^ = 0.434). In addition, a significant difference between the pain and no pain (control) condition was observed over the following 12 electrodes:

- F1 (*F*_(1,14)_ = 22.02, *p* < 0.001),
- FZ (*F*_(1,14)_ = 6.42, *p* = 0.024),
- FC1 (*F*_(1,14)_ = 33.79, *p* < 0.001),
- FCZ (*F*_(1,14)_ = 10.23, *p* = 0.006),
- C1 (*F*_(1,14)_ = 39.12, *p* < 0.001),
- CZ (*F*_(1,14)_ = 19.25, *p* = 0.001),
- CP1 (*F*_(1,14)_ = 55.31, *p* < 0.001),
- CPZ (*F*_(1,14)_ = 25.49, *p* < 0.001),
- CP2 (*F*_(1,14)_ = 8.35, *p* = 0.012),
- P1 (*F*_(1,14)_ = 33.53, *p* < 0.001),
- PZ (*F*_(1,14)_ = 42.94, *p* < 0.001),
- and P2 (*F*_(1,14)_ = 21.16, *p* < 0.001).

A significant main effect of electrodes was also observed (*F*_(14,196)_ = 29.407, *p* < 0.001, *η*^2^ = 0.677), with electrode P4 showing the maximum amplitude (*M* = 6.12 μv, *SE* = 1.09 μv) and electrode FZ showing the minimum amplitude (*M* = −2.92 μv, *SE* = 1.43 μv). Furthermore, a significant interaction between prime type and target type was also found (*F*_(1,14)_ = 21.824, *p* < 0.001, *η*^2^ = 0.609), suggesting that there was a significant priming effect of friendship on pain. Simple effect analysis showed a significant main effect of prime type (*F*_(1,14)_ = 11.546, *p* = 0.004, *η*^2^ = 0.452) with stranger prime (*M* = 1.92 μv, *SE* = 1.21 μv) eliciting a smaller LPP amplitude of pain targets than the friend prime did (*M* = 3.02 μv, *SE* = 1.24 μv). For no pain targets, there was no similar significance between stranger (*M* = 1.012 μv, *SE* = 1.34 μv) and friend (*M* = 0.477 μv, *SE* = 1.27 μv) primes (*F*_(1,14)_ = 2.374, *p* = 0.146, *η*^2^ = 0.145). Finally, no significant interaction was observed between electrodes and prime type (*F*_(14,196)_ = 0.588, *p* = 0.626, *η*^2^ = 0.040), or among electrodes, target type, and prime type (*F*_(14,196)_ = 1.300, *p* = 0.286, *η*^2^ = 0.085).

A 2 × 2 × 3 × 5 repeated measures ANOVA with prime type, target type, laterality, and anterior to posterior position of the mean ERP amplitudes recorded at the fronto–parietal electrodes, showed a significant main effect of target type (*F*_(1,14)_ = 19.255, *p* = 0.001, *η*^2^ = 0.579), suggesting that LPP was much larger in the pain condition (*M* = 2.47 μv, *SE* = 1.21 μv) than in the control condition (*M* = 0.75 μv, *SE* = 1.29 μv). There was also a main effect of anterior to posterior position (*F*_(4,56)_ = 37.240, *p* < 0.001, *η*^2^ = 0.727), a main effect of laterality (*F*_(2,28)_ = 9.850, *p* = 0.001, *η*^2^ = 0.413), and a significant interaction between target type and laterality (*F*_(2,28)_ = 30.509, *p* < 0.001, *η*^2^ = 0.685). Furthermore, there was also another interaction among target type, anterior to posterior position factor, and laterality (*F*_(1,14)_ = 5.036, *p* = 0.001, *η*^2^ = 0.265); and a significant interaction between prime type and target type (*F*_(1,14)_ = 21.824, *p* < 0.001, *η*^2^ = 0.609), suggesting a significant priming effect of friendship on pain.

#### Brain Source Estimations

**Figure 3:**
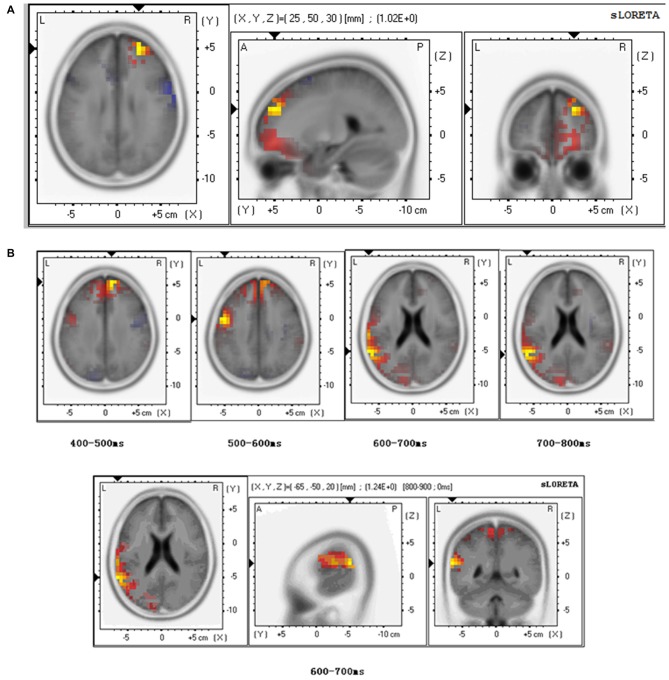
**Brain source estimation in empathy for pain. (A)** Brain source estimation of N110 in empathy for pain. Empathy for pain mainly activated the superior frontal gyrus, Brodmann 10. Red plot indicate the friend priming activation. Blue plot indicate the stranger priming activation. **(B)** Brain source estimation of LPP in empathy for pain. This component’s source varied from superior frontal gyrus (Brodmann 9) to pre-central gyrus (Brodmann 6), superior temporal gyrus (Brodmann 22), and supramarginal gyrus (Brodmann 40). Red plot indicates the friend priming activation. Blue plot indicate the stranger priming activation.

At the level of N110, sLORETA brain source estimations revealed an activation of the anterior prefrontal cortex, Brodmann 10 (Figure 3A) for both the stranger and the friend primes during empathy for pain. No significant differences were observed between these two conditions.

For LPP analysis, we conducted sLORETA brain source estimations every 100 ms between 400 ms and 800 ms post-stimulus onset. The results showed that LPP varied from superior frontal gyrus (Brodmann 9) to pre-central gyrus (Brodmann 6), superior temporal gyrus (Brodmann 22), and supramarginal gyrus (Brodmann 40; Figure 3B). A significant difference was found between the stranger and the friend prime conditions between 600–700 ms (*t* = 1.235, *p* = 0.048) in the superior temporal gyrus—the activation was stronger for the friend prime than for the stranger prime (Figure 3B).

Further sLORETA brain source estimations performed at different moment in time showed marginal significance (*t* = 1.221, *p* = 0.054) between prime type (stranger vs. friend) in Brodmann area 6 between 570–580 ms, and significant differences (*t* = 1.588, *p* = 0.047) in Brodmann area 40 between 740–750 ms (*t* = 1.588, *p* = 0.047).

#### Correlation Analyses between IRI-C and ERP from the EPT

A correlation analysis between ERP amplitude of empathy for pain and IRI-C scores revealed a significant positive correlation between the PD score (an index of subjective unhappiness when feeling others’ pain) and the ERP response to the friend prime at the level of N110 (*r* = 0.543, *p* = 0.018) and to the stranger prime (*r* = 0.593, *p* = 0.010). These correlations were observed in the pain condition, suggesting that the more the participants felt PD, the more they empathized with others’ pain, and the larger was the amplitude of N110.

At the level of LPP, our analyses also revealed a significant negative correlation between the PT score (an index of a person’s ability and motivation to adopt another person’s point of view as measured by the IRI-C, Davis, 1980) and the ERP response to the friend prime (*r* = −0.486, *p* = 0.033) and to the stranger prime in the pain condition (*r* = −0.520, *p* = 0.024). These results suggest that the more the participants took the perspective of other people, the smaller the amplitude of LPP was in response to the stranger prime or to the friend prime in the pain condition.

### Empathy for Happiness Task (EHT; Figure 4)

#### N110 (Peak Amplitude between 80–150 ms)

**Figure 4:**
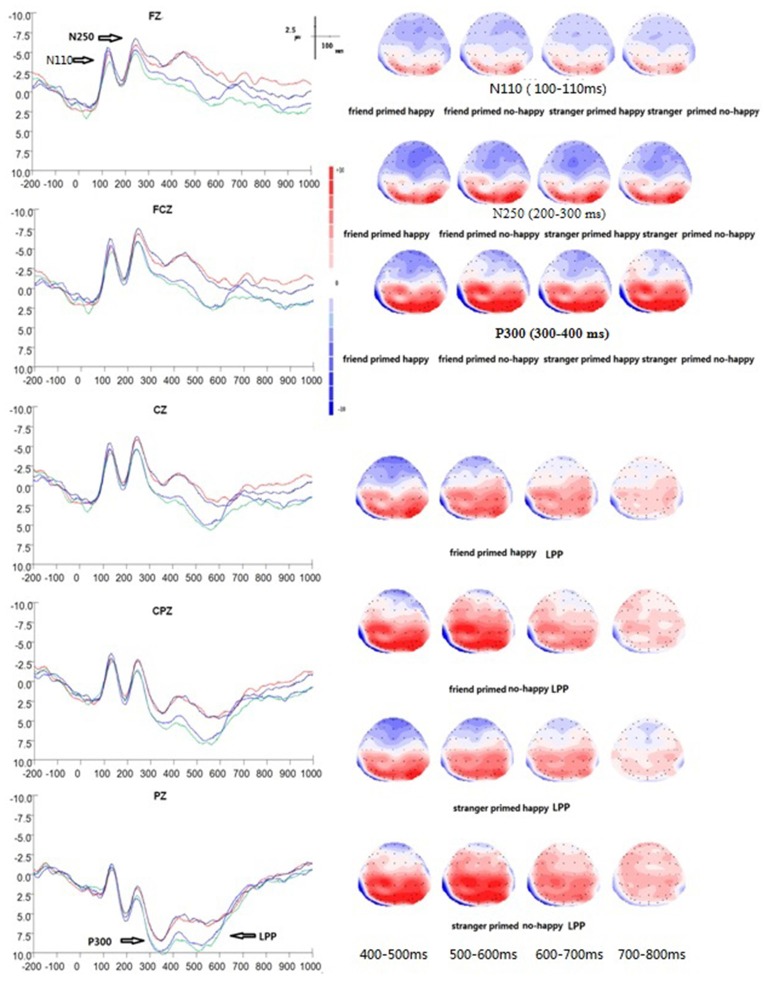
**Grand average ERP waveforms and 2D mapping of empathy for happiness.** Fz, FCz, Cz, CPz and Pz for friend prime/happy (dark blue lines), friend prime/not happy (light blue lines), stranger prime/happy (red lines), and for stranger prime/not happy (green lines).

There was a significant main effect of electrodes (*F*_(14, 196)_ = 21.364, *p* < 0.001, *η*^2^ = 0.604). No other significant effects were found over frontal-parietal areas at this early stage of information processing in EHT. No main effect of prime type (*F*_(1, 14)_ = 0.921, *p* = 0.353, *η*^2^ = 0.062), target type (*F*_(1, 14)_ = 3.855, *p* = 0.070, *η*^2^ = 0.216), or interaction was found between electrodes and prime type (*F*_(14, 196)_ = 0.751, *p* = 0.514, *η*^2^ = 0.051) or between electrodes and target type (*F*_(14, 196)_ = 2.399, *p* = 0.086, *η*^2^ = 0.146). Similarly, no significant interaction was found between prime type and target type (*F*_(1, 14)_ = 0.114, *p* = 0.741, *η*^2^ = 0.008), or among prime type, electrodes, and target type (*F*_(14, 196)_ = 0.615, *p* = 0.657, *η*^2^ = 0.042).

A 2 × 2 × 3 × 5 repeated measures ANOVA among prime type, target type, laterality, and anterior to posterior position showed, however, a main effect of anterior to posterior position (*F*_(4, 56)_ = 26.562, *p* < 0.001, *η*^2^ = 0.655), and a main effect of laterality (*F*_(2, 28)_ = 14.785, *p* < 0.001, *η*^2^ = 0.514).

#### N250 (Peak Amplitude between 200–300 ms)

In EHT, N250 peaked on average at 246 ms (*SD* = 4 ms). The ANOVAs of peak amplitude between 200–300 ms showed a significant main effect of prime type (*F*_(1, 14)_ = 6.542, *p* = 0.023, *η*^2^ = 0.318), with the stranger prime eliciting a smaller N250 (*M* = −2.72 μv, *SE* = 1.37 μv, */M/* = 2.72 μv) than the friend prime (*M* = −3.67 μv, *SE* = 1.19 μv, */M/* = 3.67 μv). Moreover, a significant effect of electrodes was found (*F*_(14, 196)_ = 28.547, *p* < 0.001, *η*^2^ = 0.671), with electrode FCZ showing the maximum amplitude (*M* = −7.93 μv, *SE* = 1.73 μv, */M/* = 7.93 μv) and electrode P4 showing the minimum amplitude (*M* = 3.73 μv, *SE* = 1.16 μv). There was no main effect of target type (*F*_(1, 14)_ = 0.952, *p* = 0.346, *η*^2^ = 0.064), and no interaction between electrodes and prime type (*F*_(14, 196)_ = 0.605, *p* = 0.629, *η*^2^ = 0.041) or between electrodes and target type (*F*_(14, 196)_ = 2.099, *p* = 0.077, *η*^2^ = 0.130). Similarly, no significant interaction was found between prime type and target type (*F*_(1, 14)_ = 0.518, *p* = 0.483, *η*^2^ = 0.036), and among prime type, electrodes, and target type (*F*_(14, 196)_ = 0.250, *p* = 0.838, *η*^2^ = 0.018).

Finally, a 2 × 2 × 3 × 5 repeated measures ANOVA revealed a main effect of anterior to posterior position (*F*_(4,56)_ = 31.918, *p* < 0.001, *η*^2^ = 0.695), a main effect of laterality (*F*_(2, 28)_ = 19.931, *p* = 0.009, *η*^2^ = 0.587), and a significant interaction between target type and anterior to posterior position (*F*_(4, 56)_ = 4.127, *p* = 0.029, *η*^2^ = 0.228).

#### P300 (Peak Amplitude between 300–400 ms)

In EHT, P300 peaked on average at 365 ms *(SD* = 18 ms). The ANOVAs of peak amplitude between 300–400 ms showed a significant main effect of prime type (*F*_(1,14)_ = 17.868, *p* = 0.001, *η*^2^ = 0.561) and electrodes (*F*_(14,196)_ = 37.552, *p* < 0.001, *η*^2^ = 0.728), respectively. The stranger prime elicited a larger P300 amplitude (*M* = 5.48 μv, *SE* = 1.46 μv) than the friend prime (*M* = 3.61 μv, *SE* = 1.31 μv). There was an interaction between prime type and electrodes (*F*_(14,196)_ = 3.535, *p* = 0.007, *η*^2^ = 0.202). Simple effect analysis also showed a significant difference between stranger and friend prime over the following three electrodes: (1) FZ (*F*_(1,14)_ = 4.94, *p* = 0.43); (2) F4 (*F*_(1,14)_ = 19.70, *p* = 0.001); and (3) FC4 (*F*_(1,14)_ = 9.17, *p* = 0.009).

At FZ, P300 was larger for the stranger’s prime (*M* = 0.44 μv, *SE* = 1.83 μv) than for the friend prime (*M* = −1.73 μv, *SE* = 1.60 μv). At F4, P300 was larger for the stranger’s prime (*M* = 0.39 μv, *SE* = 1.82 μv) than that for the friend prime (*M* = −1.20 μv, *SE* = 1.72 μv). At FC4, P300 was larger for the stranger’s prime (*M* = 1.56 μv, *SE* = 1.63 μv) than that for the friend prime (*M* = 0.48 μv, *SE* = 1.48 μv). There was no significant main effect of target type (*F*_(1,14)_ = 2.752, *p* = 0.119, *η*^2^ = 0.164), no significant interaction between target type and prime type (*F*_(1,14)_ = 0.494, *p* = 0.494, *η*^2^ = 0.034) or between target type and electrodes (*F*_(14,196)_ = 1.887, *p* = 0.134, *η*^2^ = 0.119), and no significant interaction among prime type, target type, and electrodes (*F*_(14,196)_ = 0.582, *p* = 0.645, *η*^2^ = 0.040).

Furthermore, a 2 × 2 × 3 × 5 repeated measures ANOVA among prime type, target type, laterality, and anterior to posterior position of the peak amplitude between 300–400 ms showed a significant main effect of prime type (*F*_(1,14)_ = 17.868, *p* = 0.001, *η*^2^ = 0.561), suggesting that the stranger prime elicited a larger P300 amplitude (*M* = 5.48 μv, *SE* = 1.46 μv) than the friend prime (*M* = 3.61 μv, *SE* = 1.31 μv). There were also a main effect of anterior to posterior position (*F*_(4,56)_ = 46.261, *p* < 0.001, *η*^2^ = 0.771), a main effect of laterality (*F*_(2,28)_ = 5.676, *p* = 0.009, *η*^2^ = 0.228), an interaction between prime type and laterality factor (*F*_(2,28)_ = 7.494, *p* = 0.008, *η*^2^ = 0.349), and an interaction between anterior to posterior position factor and laterality (*F*_(8,112)_ = 4.991, *p* = 0.001, *η*^2^ = 0.263).

#### LPP (Mean Amplitude at 400–800 ms)

The ANOVAs of mean amplitude at 400–800 ms showed a significant main effect of prime type (*F*_(1,14)_ = 20.582, *p* < 0.001, *η*^2^ = 0.595), with the stranger prime eliciting a larger LPP (*M* = 3.52 μv, *SE* = 0.86 μv) than the friend prime (*M* = 1.49 μv, *SE* = 0.84 μv). Also, there was a main effect of electrodes (*F*_(14,196)_ = 23.063, *p* < 0.001, *η*^2^ = 0.622), a significant interaction between prime type and target type (*F*_(14,196)_ = 4.997, *p* = 0.042, *η*^2^ = 0.263), and a significant interaction between prime type and electrodes (*F*_(14,196)_ = 10.672, *p* = 0.007, *η*^2^ = 0.433). In addition, simple effect analysis showed significant differences between stranger and friend prime over the electrode F4 (*F*_(1,14)_ = 4.69, *p* = 0.48), which indicated that LPP for stranger prime (*M* = 0.47 μv, *SE* = 1.03 μv) was significantly larger than that for the friend prime (*M* = −1.32 μv, *SE* = 1.09 μv). There was no significant main effect of target type (*F*_(1,14)_ = 0.661, *p* = 0.430, *η*^2^ = 0.045), nor an interaction between the three factors (*F*_(14,196)_ = 0.965, *p* = 0.451, *η*^2^ = 0.064).

The four-way repeated measures ANOVA of the mean amplitude at 400–800 ms showed a significant main effect of prime type, with the stranger prime eliciting a larger LPP (*M* = 3.52 μv, *SE* = 0.86 μv) than the friend prime (*M* = 1.49 μv, *SE* = 0.84 μv; *F*_(1,14)_ = 20.582, *p* < 0.001, *η*^2^ = 0.595). There were also a main effect of anterior to posterior position (*F*_(4,56)_ = 31.479, *p* < 0.001, *η*^2^ = 0.662) and a main effect of laterality (*F*_(2,28)_ = 3.822, *p* = 0.037, *η*^2^ = 0.214). There was a significant interaction between prime type, target type, and laterality (*F*_(2,28)_ = 14.893, *p* = 0.008, *η*^2^ = 0.515), and a significant interaction between anterior to posterior position and laterality (*F*_(8,112)_ = 3.104, *p* = 0.024, *η*^2^ = 0.181). Finally, a significant interaction was also found between prime type and target type (*F*_(14,196)_ = 4.997, *p* = 0.042, *η*^2^ = 0.263), between prime type and anterior to posterior position (*F*_(4,56)_ = 8.139, *p* = 0.002, *η*^2^ = 0.368), and among prime type, target type, laterality, and anterior to posterior position (*F*_(8,112)_ = 10.287, *p* < 0.001, *η*^2^ = 0.424).

#### EHT Brain Source Estimations

**Figure 5:**
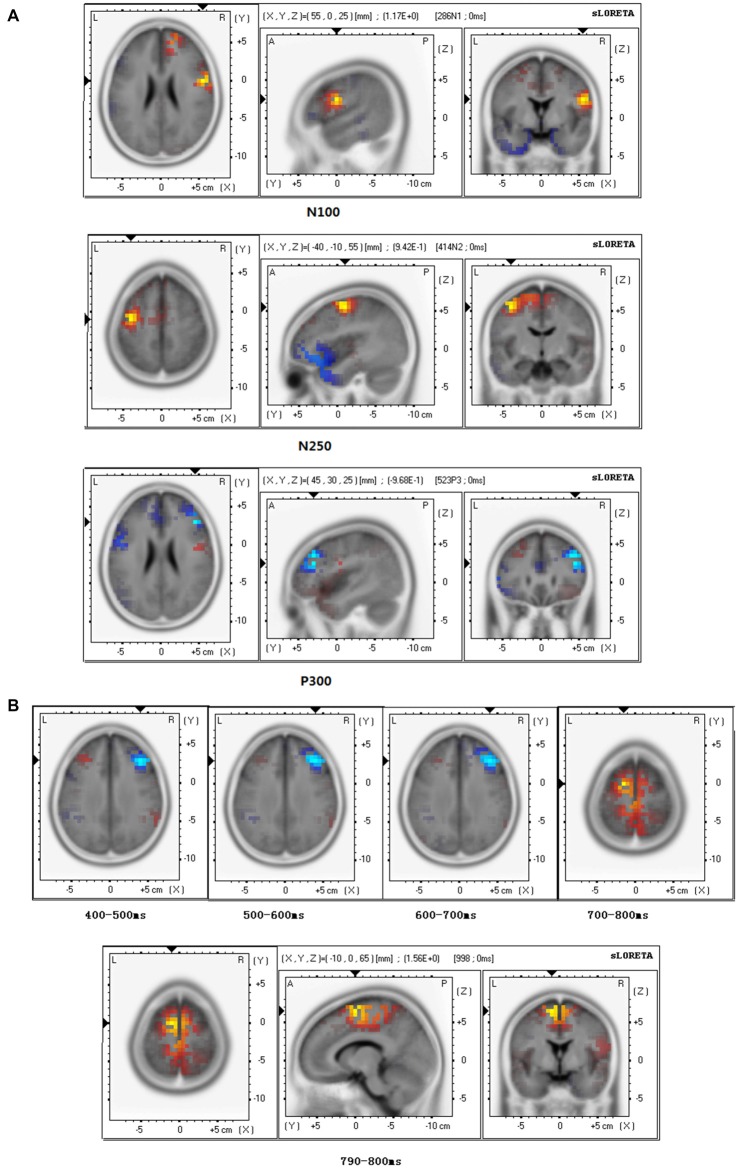
**Brain source estimation in empathy for happiness. (A)** Brain source estimations of N110, N250, P300 in empathy for happiness. Pre-central gyrus, Brodmann area 6 was activated in N110 and N250 in both conditions. For P300, both conditions mainly activated the middle frontal gyrus, Brodmann 46. Red plot indicate the friend priming activation. Blue plot indicate the stranger priming activation. **(B)** Brain source estimations of late positive potential (LPP) in empathy for happiness. LPP’s source varied from middle frontal gyrus (Brodmann area 9) during 400–700 ms to medial frontal gyrus (Brodmann area 6) during 700–800 ms. Red plot indicate the friend priming activation. Blue plot indicates the stranger priming activation.

At the N110 level and the N250 level, sLORETA brain source estimations showed that empathy for happy targets mainly activated the pre-central gyrus, Brodmann area 6 (Figure 5A), in both the stranger and the friend prime conditions. No significant differences were observed between these two conditions.

For P300, sLORETA brain source estimations showed that empathy for happy targets mainly activated the middle frontal gyrus, Brodmann area 46 (Figure 5A), in both the stranger and the friend prime conditions.

For LPP of empathy for happy targets in both the stranger and the friend prime conditions, we conducted sLORETA brain source estimations every 100 ms between 400 ms and 800 ms. Our findings showed that LPP’s brain generators varied from middle frontal gyrus (Brodmann area 9) during 400–700 ms to medial frontal gyrus (Brodmann area 6) during 700–800 ms (Figure 5B). Additional sLORETA brain source estimations performed between 790–800 ms revealed a marginal significant effect (*t* = 1.565, *p* = 0.050), showing more activation for the friend prime in the area of medial frontal gyrus activation, compared with the stranger prime.

## Discussion

The present study investigated the spatiotemporal brain dynamics of two kinds of empathy (empathy for pain and empathy for happiness) by combining electrophysiological recordings with a behavioral priming empathy task involving both negative and positive emotions. In addition, we investigated when and how these spatiotemporal dynamics were modulated by the level of interpersonal relationship between the participant and the person presented in the stimuli. Overall our results suggest that taking the perspective of a close friend (compared to that of a stranger in a priming task) has a dual-stage effect on the spatiotemporal brain dynamics of empathy. First, there is an *early* modulation for* pain*; and then there are *later* modulations *for both pain and happiness*. We discuss these early and late modulations below.

### Early Modulation of Empathy for Pain, as a Function of Interpersonal Relationship

Our study of the priming effect of interpersonal relationships on the spatiotemporal brain dynamics of empathy for pain revealed a priming effect in the early stages of information processing i.e., in a time window between 80 ms and 150 ms. This early modulation suggests an automatic attentional response related to early empathy-related brain activation, as it has been suggested in previous ERPs and magneto-encephalography (MEG) studies (e.g., Fan and Han, 2008; Han et al., 2008; Decety et al., 2010; Decety and Cacioppo, 2012). Interestingly, in our study, the fact that the N110 elicited a less negative shift for the friend prime than that of stranger prime also reinforces the assumption that a friend prime calls for more self-other overlap with the participant than the stranger primes do (Fan and Han, 2008; Ortigue et al., 2010a). This result is in line with the social comparison theory (Zhang and Zuo, 2006) and the self-expansion theory (Aron and Aron, 1996), which suggest that one shares more mental representation and emotional constructs with close friends than with strangers, due to a greater overlap of emotions and neural network activation. This, in turn, suggests easier and faster (automatic) empathic responses for close friends in pain than for strangers in pain. Interestingly, this modulation was notably observed in the anterior prefrontal cortex, a brain area known to sustain a broad variety of automatic processing, including social cognition (Amodio and Frith, 2006), bottom-up-driven processes, approach and avoidance-modulation, and evaluation-related processing (Bzdok et al., 2013).

### Late Modulation of Empathy for Pain and Happiness, as a Function of Interpersonal Relationship

Our ERP results also revealed another priming effect in later time windows i.e., the stranger prime elicited:

- a smaller LPP than the friend prime in the EPT;
- a smaller N250, a larger P300, and a larger LPP than the friend prime in the EHT.

We interpret these later differences as stimulus evaluation and classification. Overall, these later modulations occurred in brain areas involved in simulation, perspective taking, and social cognition:

- For EPT, these modulations were mainly observed in the superior frontal gyrus (Brodmann 9), pre-central gyrus (Brodmann 6), superior temporal gyrus (Brodmann 22), and supramarginal gyrus (Brodmann 40; Figure 3B). In superior temporal gyrus, activation was stronger for the friend prime than for the stranger prime (Figure 3B).
- For the EHT, these late modulations mainly occurred in the middle frontal gyrus (Brodmann 46 for P300; Figure 5A), in the middle frontal gyrus (Brodmann area 9) during 400–700 ms time window, and the medial frontal gyrus (Brodmann area 6) during 700–800 ms (for LPP; Figure 5B).

Together, these results are in line with previous studies indicating that perspective taking is a slower cognitive (and controlled) process that occurs later after a stimulus onset (Decety and Jackson, 2004; Fan and Han, 2008; Ortigue et al., 2009; Decety et al., 2010; Ortigue et al., 2010b; Decety and Cacioppo, 2012). In our study for EPT, the stranger prime elicited a smaller LPP than the friend prime in the EPT, which reinforced the assumption that one pays more attention to individuals who are self-related during empathy. During the 400–800 ms time window, LPP modulations indicate attentional process of painful cue (Polich, 2007; Dufey et al., 2011). On the other hand, we found that the P300 component had larger amplitude in response to stranger primes rather than to friend primes while judging happiness. In the EPT, the LPP evoked in response to stranger primes was smaller compared to that evoked by friend primes in pain empathy, while it showed the opposite tendency in EHT. In EHT, we also found that the P300 component had larger amplitude in response to stranger primes rather than to friend primes and a larger N250 for friend primes than for stranger primes. This finding is in line with Lamm and Lewis (2010) who suggested that N2 is sensitive to emotion processing and emotion regulation (Di Russo et al., 2006; Yuan et al., 2007; Lamm and Lewis, 2010). This EHT-related result suggests that the N250 may be sensitive to interpersonal processes during the processing of positive emotions, but not negative emotions. Further studies need to be done to further address this specific question.

### Limitations and Perspectives

The present study investigated the priming effect of interpersonal relationship on the spatiotemporal brain dynamics of two types of empathy: Empathy for pain and empathy for happiness. We combined an atypical priming paradigm with a more typical behavioral empathy task. Further studies should be done with different time intervals between the primes and targets to evaluate how the time intervals modulate our priming effects. As indicated in previous studies, a standard time interval between primes and targets for basic visual cognition, like word processing, is about 500 ms. Social cognition is, however, a more strengthening process than word processing. A shorter time interval might be sufficient to induce a similar priming effect on empathy. It has to be noted that our study had several other methodological limitations, such as a poor internal consistency of the IRI-C and a small sample size, which limits the generalization of our study.

Finally, the interpersonal relationship in our study was set by the relationship between the subjects and the prime photograph of a close friend the participants brought to the laboratory. Future studies could investigate a similar paradigm with different types of dyads (mother/child), and see how and when the priming of these different interpersonal relationships modulate the spatiotemporal brain dynamics of various types of empathy (e.g., empathy for fear, pain, happiness, and compassion).

## Author Contributions

YW designed the experiments; JS and ZZ performed the experiments; JS, FG and SY performed data analyses; SC and YW provided scientific expertise; JS wrote the original draft; SC and YW revised the entire manuscript. JS is co-first author.

## Funding

This work was supported by the National Natural Science Foundation of China [31371045] the Program for New Century Excellent Talents in Universities [NCET-11-1065], and the MOE Project of Key Research Institute of Humanities and Social Sciences at Universities [12JJD190004].

## Conflict of Interest Statement

The authors declare that the research was conducted in the absence of any commercial or financial relationships that could be construed as a potential conflict of interest.

## Footnotes

^1^http://www.uzh.ch/keyinst/NewLORETA/Private/sLORETAsetup0.exe
